# Effects of Neuromuscular Electrical Stimulation on Plantarflexors Spasticity, Gait Performance, and Self-Reported Health Outcomes in People With Chronic Stroke: A Study Protocol for a Double-Blinded Randomized Clinical Trial

**DOI:** 10.3389/fneur.2021.770784

**Published:** 2021-12-01

**Authors:** Sattam M. Almutairi, Mohamed E. Khalil, Nadiah Almutairi, Aqeel M. Alenazi

**Affiliations:** ^1^Department of Physical Therapy, College of Medical Rehabilitation, Qassim University, Buraydah, Saudi Arabia; ^2^Medical Rehabilitation Center, King Fahad Specialist Hospital, Buraydah, Saudi Arabia; ^3^College of Applied Medical Sciences, Prince Sattam Bin Abdulaziz University, Al-kharj, Saudi Arabia

**Keywords:** neuromuscular electrical stimulation (NMES), stroke, spasticity, cerebrovascular injury, waling, falls, balance

## Abstract

**Introduction:** Rehabilitation approaches have been used for people with stroke to decrease spasticity and improve functions, but little is known about the effect of neuromuscular electrical stimulation (NMES) in this population. Therefore, the primary purpose of this study was to establish a protocol for a double-blinded randomized clinical trial to examine using NMES on plantarflexors spasticity, dorsiflexor muscle strength, physical functions, and self-reported health outcomes in people with chronic stroke in Saudi Arabia.

**Material and Methods:** This randomized clinical trial with two arms and double-blinded registered in ClinicalTrials (NCT04673045) will enroll 44 participants with chronic stroke and randomized them into either the experimental group (EG), including electrical stimulation (ES) with conventional therapy or the control sham group (NMES_sham_) including placebo electrical stimulation with conventional therapy. The frequency will be set at 80 Hz for 30 min. The intervention will be three times a week for 4 weeks for both groups. Data collection for pre- and post-intervention outcomes will include measurements for the primary outcomes including paretic limb (plantarflexor spasticity, ankle range of motion, and dorsiflexor muscles strength), and gait speed using 10-m walk test (10-MWT). The secondary outcomes including mobility function using Timed Up and Go (TUG), walking endurance using 6 Minutes Walk Test (6-MWT), activity of daily living using the Arabic version of Barthel Index (BI), and self-reported health measures such as quality of life using the Medical Outcomes Survey (Short Form 36, SF-36), physical activity using Rapid Assessment of Physical Activity (RAPA), depression symptoms using Patient Health Questionnaire-9 (PHQ-9), fatigue level using Fatigue Severity Scale (FSS), and risk of fall using Fall Efficacy Scale International (FES-I). An independent *t*-test will be utilized to examine the effect of the intervention on the outcome measures.

**Results:** The recruitment has started and is ongoing.

**Conclusions:** Using 4 weeks of NMES will provide information about its effect in improving plantarflexor spasticity, dorsiflexor muscles strength, gait speed, mobility functions, and other self-reported health outcomes in people with chronic stroke when compared to NMES_sham_.

## Introduction

Stroke prevalence in Saudi Arabia has been estimated to be 0.67% of the population (1). A recent study showed that the pooled annual stroke incidence was estimated to be 29 cases per 100,000 people in Saudi Arabia (2). However, limited research in Saudi Arabia regarding the impact of stroke on functionality and other outcomes. Stroke is associated with sensorimotor impairments including spasticity, muscle weakness, and deficiency in proprioception, which are serious challenges for clinical treatment for people with stroke. Studies showed that 20–50% of stroke survivors develop spasticity after 6 months of the incidence (3, 4). Spasticity can severely limit functions and negatively impact participation in the community, activities of daily living (ADLs), and quality of life (QoL) (5).

Recently, neuromuscular electrical stimulation (NMES) has been used to inhibit spasticity (6, 7). Yang et al. (6) found that using NMES at tibialis anterior (TA) for 20 min followed by ambulation training reduced the spasticity of ankle plantarflexors. Sabut et al., (8) found that using functional electrical stimulation (FES) on the TA showed an improvement in plantarflexor spasticity by 38.3% and increased dorsiflexor muscle strength by 56.6% compared to conventional rehabilitation program (CRP) in people with stroke (8). However, there are controversial studies that did not find improvement after using electrical stimulation on the spasticity level (9, 10).

A previous meta-analysis has examined the effect of NMES on spasticity for people with stroke. This study included 29 clinical trials and found that NMES was associated with a greater reduction in spasticity. However, this study included reports with upper and lower extremities, and none of the lower extremity studies blinded the participants (11). The limitations of the previous studies were the small sample size, different stimulation time and frequency, and lacking a sham comparison group.

Using a sham NMES would have less bias and more accuracy in the findings taking into consideration the Hawthorne effect or placebo effect, especially when using electrical modalities. Furthermore, the effect of using electrical stimulation within a rehabilitation approach in Saudi Arabia is very limited, requiring further research. Thus, a treatment program with a placebo control (sham NMES, NMES_sham_) may have a better estimate on its effect on ankle plantarflexors spasticity in people with chronic stroke living in Saudi Arabia.

Therefore, the primary purpose of this study was to evaluate the effect of using NMES on spasticity of the ankle plantarflexors, ankle range of motion (ROM), dorsiflexors strength, and gait speed in people with chronic stroke. The secondary purpose of this study was to explore the effect of using NMES on mobility function and physical performance measures including walking endurance and ADL and self-reported health measures including QoL, physical activity, depression symptoms, fatigue, and risk of fall in this population.

## Materials and Methods

The protocol was developed according to the Standard Protocol Items: Recommendations for Interventional Trials (SPIRIT) guidelines and checklists.

### Study Design

This study is a two-arm, parallel, double-blinded, single-site, randomized clinical trial (RCT) with two independent variables, between-subject factor (group), and within-subject factor (time). The independent variable of groups has two levels: active NMES (experimental group) and placebo group. The independent variable of time has two levels: baseline (T_0_) and post-intervention (T_1_). The participants will be assigned randomly on a 1:1 ratio into either the experimental group (EG) or to the placebo group (NMES_sham_). Outcomes assessor and participants are blinded to the allocation of groups.

### Study Setting

This study will be conducted at the physical therapy department, King Fahad Specialist Hospital, Buraydah, Saudi Arabia.

### Registry of Clinical Trials

The study protocol was registered in ClinicalTrials (registration number NCT0467304).

### Participants and Recruitment

**Figure 1:**
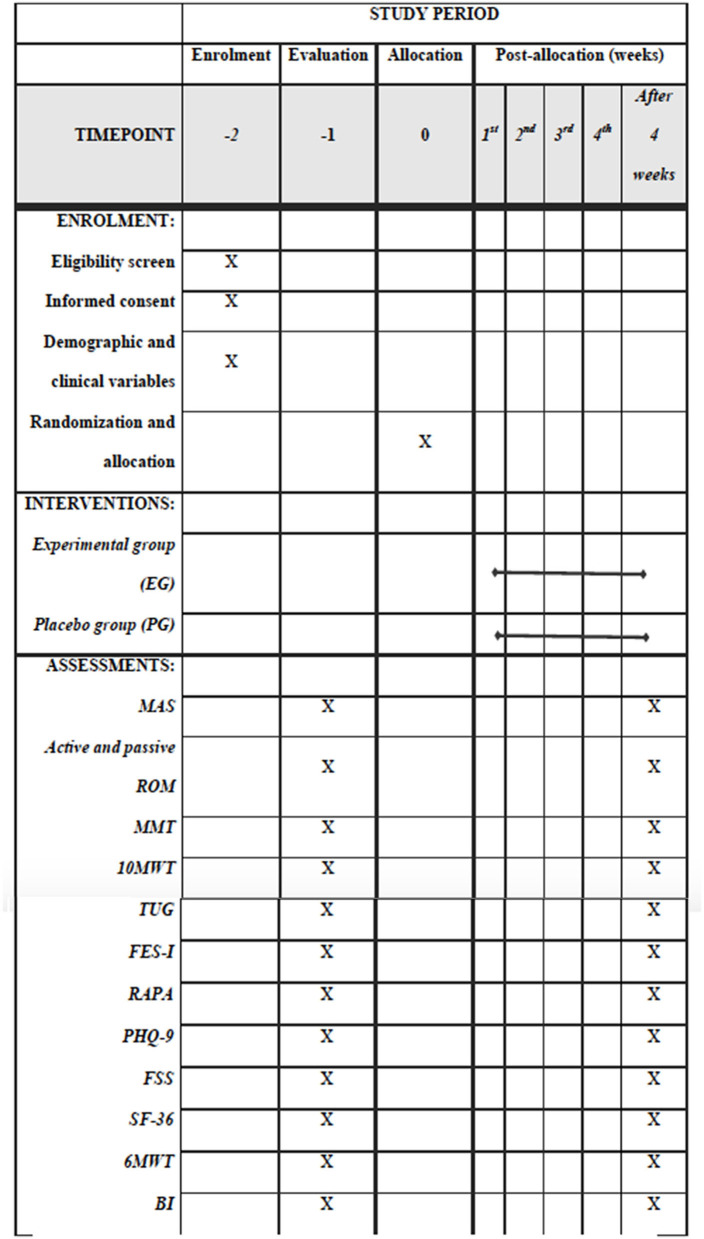
Standard Protocol Items: Recommendations for Interventional Trials (SPIRIT) for the content of the schedule of enrolment, interventions, and assessments checklist.

About 44 men and women of any race or ethnicity with a brain stroke will be recruited in the Qassim area to participate in this study based on a previous study (7). The participants will be recruited through advertising in the local communities, health professionals, family members, and support groups. Prior to any testing or data collection, all of the participants will read and sign an informed consent form describing this study approved by national bioethics committee review boards in Saudi Arabia. Upon entry into the study, the principal investigator (PI) or the co-investigators will explain the research project to the participants and inform them of the risks and benefits of the study. Then, a questionnaire of demographic and medical history information will be obtained. Participants will be screened by an experienced assessor for eligibility to participate in this study. The timepoints for recruitment, assessments, and interventions are shown in Figure 1.

#### Ethical Approval

The study protocol is approved by the national bioethics committee review boards of the Ministry of Health, Saudi Arabia (1442-551803). All participants give written informed consent prior to the study enrollment.

#### Inclusion Criteria

Participants will be included if they had a first incident of brain stroke aged from 18 to 65 years old and have at least 6 months since stroke to avoid the effects of spontaneous recovery. Independent ambulatory ability with or without assistive device at least 10 m (e.g., cane and walker), plantarflexors spasticity on paretic limb ≥2 on modified Ashworth scale (MAS) (12), and functional ambulation ≥3 on functional ambulation categories (13).

#### Exclusion Criteria

Participants will be excluded from the study if they did not meet the inclusion criteria. Furthermore, participants will be excluded if they have skin integrity issues on contact surface of NMES, significant cognitive impairments (unable to follow three step commands), other serious medical conditions, history of other neurologic or orthopedic disorder affecting walking function, more than one previous stroke, contraindications to NMES, such as a pacemaker or tumor, injected with any medicine that reduce spasticity (e.g., Botulinum-A Toxin), pregnancy, and previous lower limb treatment with FES or NMES.

### Outcome Measures

Primary and secondary outcome measures will be collected by a blinded trained assessor at the baseline and after the intervention (4 weeks of intervention). Primary outcomes will include spasticity in the plantarflexor muscles in paretic leg measured by MAS, active and passive ROM for the ankle joint in paretic leg measured by a handheld goniometer, muscle strength for the ankle dorsiflexor muscle in paretic leg measured by manual muscle testing (MMT), gait speed using 10-meter walk test (10-MWT). Secondary outcome measures will include mobility function using Timed Up and Go (TUG), walking endurance using 6 MWT, ADL using the Arabic version Barthel index (BI), and self-reported health measures such as Medical Outcomes Survey (Short Form-36, SF-36), physical activity using RAPA, depression symptoms using Patient Health Questionnaire-9 (PHQ-9), fatigue level using the Arabic version of Fatigue Severity Scale (FSS), and risk of fall using the Arabic version of Fall Efficacy Scale International (FES-I).

#### Primary Outcome Measure Procedures

##### Demographic and Clinical Variables

The diagnosis, age, sex, marital status, education, employment, self-reported comorbidities such as diabetes and hypertension, self-reported medications, self-reported history of falls in the past 12 months, self-reported pain in the past month, and body mass index using weight (kg) and height in (cm) will be obtained at the baseline session. The diagnosis of stroke includes location of injury, stroke type, artery infarction, side of the body paralysis, dominant side, and duration of the stroke.

##### Modified Ashworth Scale

The spasticity in the plantarflexor muscles tone will be measured on the affected leg using MAS. Participants will be placed in a supine position. To test ankle plantarflexor muscles' spasticity, from maximal ankle plantarflexor position, the assessor passively moves the ankle to maximal dorsiflexion position over 1 s. The test will be performed at the baseline and post-intervention. Spasticity will be graded according to MAS, which is a 6-point rating scale (12):

- 0 No increase in muscle tone.
- 1 Slight increase in muscle tone, manifested by a catch and release or by minimal resistance at the end of the ROM when the affected part(s) is moved in flexion or extension.
- 1+ Slight increase in muscle tone, manifested by a catch, followed by minimal resistance throughout the remainder (less than half) of the ROM.
- 2 More marked increase in muscle tone through most of the ROM, but affected part(s) easily moved.
- 3 Considerable increase in muscle tone, passive movement difficult.
- 4 Affected part(s) rigid in flexion or extension.

##### Active and Passive Range of Motion

The test consists of active and passive ankle joint ROM. The measurement will be in degrees using a handheld goniometer. Goniometry will be performed with the subject in a supine position with extended knees, and the measurement will be made at the neutral position between dorsal flexion and plantar flexion. The axis of the goniometer will be placed 2 cm below the medial malleolus, and its moving axis will be placed along the first metatarsal bone. The passive ROM will be determined as the range that the assessor is able to move the subject's ankle beginning in maximum plantarflexion to maximum dorsiflexion until any resistance is felt. Similarly, the active ROM will be measured by asking the participants to move joints maximally. The test will be performed at the baseline and post-intervention. Three measurements for the active and passive ROM will be taken, and the average will be calculated for the ankle dorsiflexion ROM.

##### Manual Muscle Testing for Ankle Dorsiflexors

In clinical practice, muscle strength is most often evaluated using the manual muscle strength testing of the Medical Research Council (MRC) grade. The ankle dorsiflexor strength will be graded according to the (MMT; graded from 0 (no contraction at all) to 5 (full range of movement against power and the same force as on the opposite side) for ankle dorsiflexor. The test will be performed at the baseline and post-intervention. To perform the test, the participant will be in short sitting with ankle plantarflexed. The assessor will be in front of the participant sitting using one hand to stabilize the leg just above the malleoli. The other hand will provide the resistance to the dorsal aspect of the foot. A participant will be asked to dorsiflex his/her ankle actively against the resistance (14).

##### 10-Meter Walk Test

The 10-MWT assesses self-selected preferred walking speed over a short duration with or without an assistive device. The participant will be asked to walk a total of 10 m where an acceleration zone is used for the participants to accelerate 2 m before entering the 6-m distance and 2 m to decelerate afterward. Speed is only calculated for the 6-m distance between the end zones. The 10-MWT is widely used in clinical practice and research for people with stroke and has been shown to have an excellent test–retest reliability (ICC > 0.95) (15). The minimal clinically important difference (MCID) is 0.14 m/s for substantial meaningful change (16). The test will be performed three times, and the resulting speeds obtained will be averaged and used for the analysis. The test will be performed at the baseline and post-intervention.

#### Secondary Outcome Measures Procedures

##### Timed Up and Go

The TUG assesses functional mobility by assessing an individual's ability to stand up, walk 3 m at a comfortable pace, turn 180**°**, walk back 3 m, and sit down (17). The TUG test has excellent reliability and validity in stroke population, and the minimal detectable change (MDC) is 2.9 s (18). **Two** practice trials of the TUG will be allowed to familiarize the participant with the task. TUG is a valid method for screening of functional mobility and risk for falls in community-dwelling elderly people (17).

##### Six-Minute Walk Test

It assesses the distance walked over 6 min as a test of aerobic capacity and endurance. In this test, the patient can have standing rest as many as they like, but the timer should keep recording. The number of rests taken, and the time will be documented. In addition, the patient can use any assistive device or braces and will be documented. Only minimum amount of assistance is accepted if the patient needs it, and the level of the assistance should be documented. The examiner should walk behind the patient at least half a step when the patient administering the test. Turnaround points are to be marked by a cone. The participants will be asked to eat a light meal and wear comfortable clothes and shoes. Participants will be informed every minute elapsed. The heart rate, blood pressure, and oxygen saturation will be taken before and after the test.

##### The Barthel Index

It contains 10 common ADL to assess disability (19). It includes feeding, grooming, bathing, dressing, bowel and bladder care, toilet use, ambulation, transfers, and stair climbing. The scale yields a total score out of 100. The higher the score, the greater the degree of functional independence. The MCID is 1.85 in stroke population (20). The BI has been demonstrated to be a useful instrument with high inter-rater reliability, internal consistency, convergent and predictive validity, and adequate responsiveness in stroke patients (21). The ADL performance of each patient will be rated primarily by interviewing the patients, their primary caregiver, or their nurse. Observation of performance will be applied if necessary. The BI has been translated into the Arabic language following the international guideline.

##### Medical Outcomes Survey (SF-36)

It is a health survey that evaluates QoL in clinical practice and research purposes. It has eight-dimensional subscales: physical functioning to assess physical activities limitation (e.g., lifting heavy objects, playing golf, climbing several flights of stairs, walking more than a mile, and bathing or dressing), role limitations due to physical health problems (e.g., difficulty performing the work or the daily activities), general health perceptions, vitality, social functioning (e.g., limitation in social activities due to physical or emotional problems), role limitations due to emotional problems (e.g., limitation in daily activities at work or other activities due to emotional problems), general mental health, and health transition. Answers will be in different scales with different anchor points. Total scores will range from 0 indicating the worst QoL to 100 indicating the best QoL. Thus, a higher score indicates a higher self-perceived QoL. SF-36 has been translated and validated into Arabic language (22, 23).

##### Rapid Assessment of Physical Activity

It is a 9-item, self-reported questionnaire that measures the levels of physical activity of adults older than 50 years. The response to each item is yes or no. The instructions for completing the questionnaire provide a brief description of three levels of physical activity (light, moderate, and vigorous) with graphic and text descriptions of the types of activities that fall into each category. The total score of the first seven items is from 1 to 7 points, with the score of a respondent categorized into one of five levels of physical activity: 1 = sedentary, 2 = underactive, 3 = regular underactive (light activities), 4 = regular underactive, and 5 = regular active. Responses to the strength training and flexibility items are scored separately, with strength training = 1, flexibility = 2, or both = 3 (24). The RAPA measure has been cross-culturally adapted and validated to the Arabic language (25).

##### Patient Health Questionnaire-9

It is an instrument to evaluate depression symptoms among different populations. It has nine items, and each item uses a Likert scale of four options to rate the depressive symptoms based on frequency and occurrence in the past 2 weeks such at:

- 0 not at all
- 1 several days
- 2 more than half the days
- 3 nearly every day

A total score of 5–9 indicates minimal symptoms, 10–14 indicates moderate symptoms, 15–19 indicates major symptoms, and more than 20 indicates severe depression (26–28). Previous research reported it as a reliable and valid instrument for different populations including people with stroke (29). This instrument has been translated and validated into different languages including Arabic language (30, 31).

##### Fatigue Severity Scale

It is a self-reported questionnaire that consists of **nine** statements that rate the severity of the fatigue interference of the patient with certain activities. The items are scored from 1 to 7 with 1 = strongly disagree and 7 = strongly agree. The minimum score = 9 and the maximum score possible = 63. The higher score indicates greater fatigue severity (32). The mean score of the **nine** items will be used for statistical analysis. The FSS has been shown to have high internal consistency, good test–retest reliability, and good concurrent validity in several populations (33–35). The FSS scale has been translated and validated into the Arabic language (36).

##### Fall Efficacy Scale International

The Fall Efficacy Scale International has been used to assess the risk of fall in older adults and people with chronic conditions (37). It is a 16-item self-reported questionnaire. Each item involves an activity that scored by the participant using the 4-point Likert scale depending on how concerned to fall if they did this activity regardless of actual performance. The score ranges from 16 to 19 indicating low concern about fall, 20 to 27 indicating moderate concern, and 28 to 64 indicating a high risk of fall. This scale has been translated and validated into Arabic language (38).

### Procedure

#### Assignment of Intervention

##### Allocation Sequence Generation

The participants will be randomly allocated at a 1:1 ratio into either EG (*n* = 22) or NMES_sham_ (*n* = 22). The randomization process will be generated by an independent research assistant who is not involved in the treatment or data collection using an online randomization website (https://www.graphpad.com/quickcalcs/randomize1.cfm).

##### Allocation Concealment

All randomized allocations of participants will be placed in a sealed envelope for each participant with no further identification. A research assistant will prepare envelopes and withhold information from assessors and participants. After completing the baseline assessment, a research assistant will draw an envelope and inform a trained therapist who is not involved in the study about the allocation of patients.

##### Blinding

In this double-blind study, the assessor and participants will be blinded to the allocation of group. The assessor will be banned from attending interventional sessions for both groups, and the allocation of participants will be managed in schedules to minimize contact between participants in both groups.

#### Intervention

After the informed consent, participants will complete an intake form for demographic data, past medical history, past surgical history, and activity level. Additionally, they will be screened for inclusion/exclusion criteria. Participants who meet the inclusion/exclusion criteria will be enrolled to the study and evaluated on the main outcomes prior to and after the intervention. The participants will be randomized using the above-mentioned method in allocation sequence generation and located in two groups.

All participants will receive a conventional rehabilitation program (CRP) including warming up, strengthening, stretching exercise, balance exercise, and gait training for 45 min/day, three times a week for 4 weeks (Table 1). In addition, EG will receive 30 min active NMES, and the placebo group will receive 30-min NMES_sham_. There is a variety in the duration of using the NMES in the literature from a few minutes to hours (39–41). However, 30 min of electrical stimulation is an acceptable time for the clinicians and patients in the clinical sitting, and to keep the adherence of the patient at the maximum level to the treatment program.

**Table 1:** Description of conventional rehabilitation program components.

**Components**	**Time** **repetitions**	**Description**
Warming up	5 min	Bicycling using stationary bicycle or ergometer
Stretching exercise	5 min 3 × 10 s with 10-s rest	Unilateral for the following muscle groups: wrist flexors, biceps, pectoral major, shoulder extensors, quadriceps, hamstrings, gastrocnemius, and thigh adductors
Strengthening exercise	5 min 3 × 40 repetitions with 10-s rest 3 × 40 repetitions with 10-s rest	Upper extremity strengthening exercises using small pulley weight Lower extremity strengthening exercise using quadriceps chair
Postural control and balance	3 min 3 × 50 s with 10-s rest 3 min 3 × 50 s with 10-s rest 4 min 4 × 50 s with 10-s rest	Sit to stand transition with symmetrical weight bearing and trunk rotationDynamic balance activity includes low frequency sway and increase weight shifting on the affected side Gentle perturbations to displace COG using gymnastic ball or equilibrium
Upper extremity control	5 min 3 × 10 repetitions with 10-s rest	Moving the upper extremity with emphasis on scapular motion. For example, hand to mouth, hand to opposite side, and hand functions Grasping and releasing objective
Lower extremity control	3 min 3 × 10 s with 10-s rest	Pre-gait mat activity includes hook lying, bridging, and lower trunk rotation
Gait training	12 min 12 × 30 s with 30-s rest	Gait training using parallel bar. Gait training includes forward, backward, sideward step, and in crossed pattern

The equipment Gymna (Pasweg 6a, 3740 Bilzen, Belgium) will be used for active and placebo Russian electrical stimulation therapy. A frequency up to 100 Hz is acceptable for treatment and produces muscle contraction in a clinical setting. Low frequency produces low muscle contraction (twitches or a tremor). As the frequency increases, the muscle contraction increases and, therefore; muscle strength increases (42). However, we will select 80 Hz to stimulate dorsiflexor muscle strength without getting the risk of getting the maximum high frequency. Table 2 shows the parameters that will be used in the active electrical stimulation.

**Table 2:** Parameters for active electrical stimulation.

Carrier wave	2.5 kHz
Burst	80 Hz
On time	5 s
Off time	15 s
Treatment time	30 min
Current type	Constant current
Waveform	Sinusoidal

**Figure 2:**
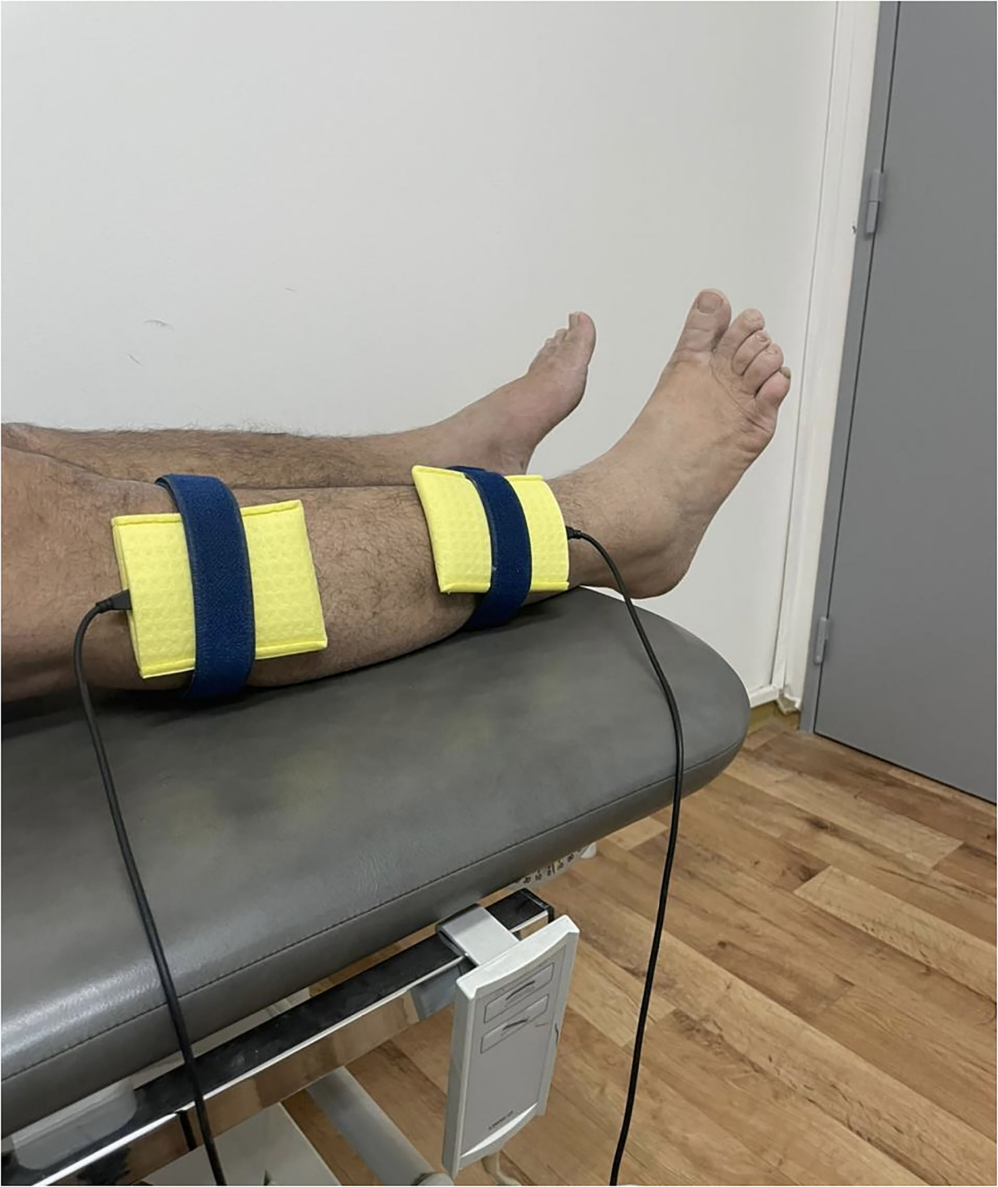
The placement of electrodes on the dorsiflexor muscles of patient with hemiplegia.

The NMES delivered the electrical current through two surface electrodes (6 × 8 cm) inserted in saline-soaked sponges. The intensity of stimulation will be set within the tolerance level of the subject. The amplitude will be adjusted to produce visible ankle dorsiflexor muscle contraction without affecting the comfort of the patient. The cathode electrode will be placed over the common peroneal nerve as it passes over the head of the fibula, and the anode will be placed on the middle of the ankle dorsiflexor muscle belly on one-third of the line between the fibular head and the medial malleolus on the paretic limb (see Figure 2).

During the stimulation, the participants will be instructed to remain in the supine position and relax. For the placebo group, electrodes will be placed at the same position as the active NMES condition; however, the current intensity will be gradually decreased after a few seconds to 0. Therefore, the participant will experience a passage of current on the muscle at the beginning but receive no current for the rest of the stimulation period. The participants will be informed that the stimulation is below the sensory level. After each visit, the participants would be investigated for any pain or skin irritation under the electrode. Adverse events would be listed and discussed with the participant. The pre- and post-training assessments will be completed within 3 days before and after the training sessions.

#### Sample Size Calculation

The sample size was calculated based on a previous study (7). However, considering a 10% attrition rate, the total sample size will be 44 participants. Each group had 22 participants.

### Statistical Analysis

IBM SPSS Version 25.0 (IBM Corp., Armonk, NY, USA) will be used to analyze the collected variables. The descriptive demographic information (means, medians, and SD) include age, gender, onset of the stroke, height, weight, and all outcome measures will be calculated and compared at the baseline using the independent *t*-test or chi-square test to ensure that there is no significant difference between the two groups at the baseline. Normality of the data will be assessed using the Shapiro–Wilk test. Comparisons between pre- and post-intervention will be conducted using the dependent *t*-test or the Wilcoxon signed rank test to determine if there will be significant differences between two different timepoints for each group. The independent *t*-test or the Mann–Whitney *U*-test will be used to compare the recorded values at the post-intervention between the two groups. In case of confounding variables such as age, secondary analysis will be performed according to the age groups (18–30, 31–50, and 51–65 years). In order to account for pre- and post-intervention between-group clinical effectiveness in terms of the outcome measures, Cohen's *d* effect size will be calculated. Furthermore, the pre-test effect sizes will be subtracted from the post-test effect sizes to effectively control for the differences at the baseline and obtain the absolute effect size. Effect sizes will be defined as small (*d* ≤ 0.2), medium (0.2 > *d* < 0.8), and large (*d* ≥ 0.8) (43). Differences will be considered statistically significant when *p* < 0.05.

### Research Training

Physical therapists have taken training sessions on delivering the intervention and using NMES. The blinded assessor was trained on data collection and measuring all outcomes for participants.

### Data Management

All participants will be informed and assured that all identifiable data from potential and enrolled participants will be maintained confidential before, during, and after the study by encoding the name of participants. All data will be stored in a lockable cabinet at KFSH in individual numbered participant files. The files will be saved in numerical order and have restricted access. The consent form copies will be saved in a separate cabinet since it has identifiable information and restricted access. All data will be checked regularly for completeness and validity prior to data entry. The outcomes data will not be checked by the therapist who is applying the intervention.

## Discussion

The main objective of this clinical trial is to evaluate the effect of using NMES on plantarflexor muscle spasticity, ankle ROM, dorsiflexor muscle strength, and gait speed. The secondary objective of this work is to examine the effect of using NMES on mobility function, walking endurance, and self-reported outcomes such as ADL, QoL, physical activity, depression symptoms, fatigue level, and risk of fall.

The findings from this study will highlight the importance and effect of using electrical stimulation during a rehabilitation program for individuals with chronic stroke living in Saudi Arabia. If the findings are positive, this study will be the first that has preliminary positive results for a rehabilitation program using NMES in this population in Saudi Arabia. The results will eventually help healthcare providers and other researchers in choosing and investigating the appropriate rehabilitation approach to improve spasticity and functionality.

Upon completion of data collection, it is expected that the active NMES group will benefit from decreased ankle plantarflexors spasticity, increased dorsiflexors strength, and improved walking function, physical activity, and QoL. The placebo group will gain benefit from CRP; however, it is expected that the scores of placebo group in targeted outcomes will show less variation than EG. The result will be published after the data and the study is completed.

Although this study has some strengths including blinded assessors, blinded participants, and placebo NMES to decrease bias, there are some limitations. A single center study is a possible limitation that may limit the generalizability of the results. Using a clinical setting (hospital outpatient clinic) with lack of tools is another limitation that may limit the use of objective tools such as EMG. Drop out is another limitation that may occur in clinical trials that affects sample size and results. Lack of follow-up assessment after the intervention is another limitation due to limited financial resources that affect the results of long-term effect and persistence of the results of this treatment.

## Ethics Statement

The studies involving human participants were reviewed and approved by the National Bioethics Committee Review Boards of Ministry of Health. The patients/participants provided their written informed consent to participate in this study.

## Author Contributions

SA and AA drafted the manuscript proposal, designed the study, acquired the data and were involved in sorting, and analyzing data. MK and NA participated in the recruitment of patients and took outcome measures. All authors are involved in interpreting the data and drafting the final manuscript.

## Funding

The study was funded by Qassim University with ID (10126-fcohsb-2020-1-3-I) on October 13, 2020. The funding source had no role in the study design, data analysis, reporting the result, or interpretation of data, writing of reports, and submission for publication.

## Conflict of Interest

The authors declare that the research was conducted in the absence of any commercial or financial relationships that could be construed as a potential conflict of interest.

## Publisher's Note

All claims expressed in this article are solely those of the authors and do not necessarily represent those of their affiliated organizations, or those of the publisher, the editors and the reviewers. Any product that may be evaluated in this article, or claim that may be made by its manufacturer, is not guaranteed or endorsed by the publisher.
